# Efficacy and outcomes of inclisiran in the management of homozygous and heterozygous familial hypercholesterolemia: a systematic review and meta-analysis

**DOI:** 10.1097/MS9.0000000000002982

**Published:** 2025-02-06

**Authors:** Zaraq Khan, Ajeet Singh, Muhammad Haseeb Ur Rasool, Nimra Qasim, Muhammad Yafaa Naveed Chaudhary, Saif Syed, Monazza Riaz, Wajih Rehman, Ihtiham Rehman, Rabia Islam, Hamza Islam, Aqeel Muhammad, Raveena Devi, Calvin R. WEI, Farheen Naaz, Aymar Akilimali

**Affiliations:** a Indiana University Southwest Internal Medicine, Evansville, Indiana, USA; b Dow University of Health Sciences, Karachi, Pakistan; c Icahn School of Medicine at Mount SINAI, New York, New York, USA; d Peoples University of Medical and Health Sciences for Women, Sindh, Pakistan; e RCSI Dublin, Dublin, Ireland; f Norfolk and Suffolk NHS Foundation Trust, Norwich, United Kingdom; g Saidu Medical College, Khyber Medical University, KPK, Saidu Sharif, Pakistan; h Punjab Medical College, Faisalabad, Pakistan; i Isra University, Hyderabad, Pakistan; j Department of research and development, Shing Huei Group, Taipei, Taiwan; k Medical college, Deccan College of Medical Sciences, Hyderabad, India; l Department of Research, Medical Research Circle (MedReC), Goma, DR Congo

**Keywords:** heterozygous, homozygous, hypercholesterolemia, inclisiran, pcsk9 inhibitor

## Abstract

**Objective:**

To find out the efficacy of Inclisiran sodium in heterozygous familial hypercholesterolemia (HeFH) and homozygous familial hypercholesterolemia (HoFH) patient groups.

**Methods:**

PubMed, Embase, and Clinicaltrials.gov databases were searched for the relevant studies. Atherosclerotic parameters considered included LDL-C, total cholesterol, PCSK9, apolipoprotein-B, and non-HDL-C. Primary outcomes were the percentage difference in atherosclerotic parameters at follow-up relative to baseline values. Our study examined these primary outcomes to determine whether there is a statistically significant difference between the HeFH and HoFH groups. The risk of bias was assessed by the Cochrane risk of bias tool.

**Results:**

Analysis of four ORION clinical trials unveiled the significant difference in pooled mean differences in LDL-C (HeFH: –48.62%, HoFH: –9.12%; *P* < 0.05) total cholesterol (HeFH: –30.31%, HoFH: –11.50%; *P* < 0.05), apolipoprotein-B (HeFH: –39.97%, HoFH: –14.68%, *P* < 0.05), and non-HDL-C (HeFH: –44.51%, HoFH: –12.22%; *P* <0.05) between HeFH and HoFH groups. However, the difference in the pooled mean difference in PCSK9 values (HeFH: –68.41%, HoFH: –56.25%; *P* = 0.2) between the HeFH and HoFH groups was statistically insignificant. Studies were of high quality.

**Conclusion:**

There was a significant difference in the reductions in atherosclerotic lipid parameters in heterozygous and homozygous populations after the administration of inclisiran except for the PCSK9 parameter. Further studies are needed to support this conclusion.

## Plain language summary

The purpose of this study was to evaluate whether inclisiran sodium has differing levels of effectiveness in treating two distinct forms of familial hypercholesterolemia: homozygous (HoFH) and heterozygous (HeFH). These conditions significantly impact cholesterol levels and risk for cardiovascular disease. To conduct this analysis, extensive searches were performed across several databases, including Clinicaltrials.gov, Embase, PubMed, and ORION clinical trials, aiming to gather relevant data for a systematic review and meta-analysis. The study focused on atherosclerotic markers such as non-HDL-C, PCSK9, total cholesterol, LDL-C, and apolipoprotein-B. Changes in these markers from baseline to follow-up were critically analyzed to assess the treatment’s impact. The primary measurement was the percentage change in these atherosclerotic parameters. The Cochrane risk of bias tool was employed to ensure the integrity of the study findings. Meta-analyses were conducted when at least two studies reported outcomes for the same variable. Data from four ORION clinical studies were included, providing a robust foundation for evaluating inclisiran sodium’s comparative effectiveness across patient groups with HoFH and HeFH.

## Introduction

The most prevalent hereditary metabolic illness, familial hypercholesterolemia (FH), is caused by mutations in genes affecting the metabolism of low-density lipoprotein cholesterol (LDL-C)^[1–3]^. Because it is an autosomal dominant condition, both carriers, or homozygotes and heterozygotes, are impacted. Those who are homozygous have severe disease. Heterozygous familial hypercholesterolemia (HeFH) and homozygous hypercholesterolemia (HoFH) have different prevalences. A recent meta-analysis reported the average worldwide prevalence of HeFH to be at least 1 in 300^[4]^. Prior estimates of the prevalence of HoFH were 1 in 1 000 000, but more recent surveys using genetic analysis suggest 1 in 170 000 to 300 000 cases.^[5]^

Inclisiran is a small-interfering RNA (siRNA) drug that targets the mRNA of the PCSK9 gene to reduce the synthesis of intracellular and extracellular PCSK9^[2]^. It is a double-stranded siRNA that binds and then degrades the specific messenger RNA (mRNA), thus preventing its translation into the PCSK9 protein^[3]^. This results in the downregulation of LDL-C receptor lysosomal catabolism, supporting more efficient clearance of LDL from the bloodstream^[3]^. Therefore, this drug proves to be a great therapeutic agent for familial hypercholesterolemia.

**HIGHLIGHTS**

- Inclisiran is a novel drug being studied for its efficacy in lowering atherogenic lipid parameters in people with hypercholesterolemia.
- Familial hypercholesterolemia has different prognosis in patients, depending on the genotype inherited, and multiple ORION trials indicate different efficacy of inclisiran in patients having heterozygous and homozygous familial hypercholesterolemia.
- No previous research was done to study and compare the effect of inclisiran on atherogenic parameters in patients with heterozygous and homozygous traits for hypercholesterolemia.
- This research unveils the different efficacy of inclisiran in patients with heterozygous and homozygous familial hypercholesterolemia.
- This increases the scope of knowledge regarding the efficacy of this drug and helps clinicians apply this knowledge into practice.

Multiple clinical studies were designed to assess the efficacy, safety, and tolerability of long-term dosing of inclisiran given as subcutaneous injections in participants with HeFH or HoFH, having high cardiovascular risk and elevated low-density lipoprotein cholesterol (LDL-C). Among these studies, ORION trials were conducted to provide important data regarding the efficacy and tolerability of inclisiran. In these trials, patients were enrolled having either HeFH or HoFH characterized by elevated levels of low-density lipoprotein (LDL) cholesterol.

These trials report different efficacies of inclisiran in patients having either HeFH or HoFH. No meta-analysis has been conducted to date to compare the efficacy of this drug in patients having HoFH and HeFH. Hence, our systematic review and meta-analysis aims to compare the efficacy of inclisiran in HoFH and HeFH.

## Methods

We conducted the systematic review and meta-analysis according to the Preferred Reporting Items for Systematic Reviews and Meta-Analyses (PRISMA) statement. The study is registered in Open Science Forum. The protocol of this study was submitted on the PROSPERO with the registration code yet to be received.

### Data source and search strategy

We conducted a broad literature search to identify ORION clinical trials comparing inclisiran to placebo drugs. Two independent authors (M.Y.N.C. and Z.K.) conducted a comprehensive search for clinical trials published up until 3 July 2023, using the databases Clinicaltrials.gov, Embase, and PubMed to evaluate the evidence for this goal. The electronic search strategy included both Medical Subject Headings (MeSH) and keywords (free text words). Search terms included the keyword terms: “Inclisiran,” “Inclisiran Sodium,” “Hypercholesterolemia,” “ORION Trials,” and “PCSK9 inhibitors.” Any discrepancy (if present) was resolved by a third author (M.H.R). Only human participants and English-language articles were included in the study. Endnote X7 software was used to manage and eliminate duplicates from this literature. Reference lists and topic-related reviews were checked manually to identify further relevant papers.

### Study selection and inclusion/exclusion criteria

We used the PICO (Patient/Population, Intervention, Comparison, and Outcomes) framework to establish the following inclusion criteria for all relevant original articles: Population of familial hypercholesterolemia; intervention of 300 mg inclisiran; comparator of HoFH and HeFH; and outcomes of change in apolipoprotein-B levels, LDL-C levels, non-HDL levels, total cholesterol, and PCSK9 levels. We included studies with specified familial hypercholesterolemia only (either HeFH or HoFH). Studies reporting familial hypercholesterolemia with unspecified genotypes were excluded. Research studies that were case reports, reviews, letters, or conference abstracts without the complete text were excluded. Two authors (N.Q. and M.Y.N.C.) screened the titles and abstracts for full texts. Any disagreements were resolved by the fourth author (M.J.). All included studies represented unique trials. The inclusion criteria for ORION trials are given in Table 2.

### Data extraction and outcome measure

Data extraction was done from all eligible articles. Primary outcomes included the five atherosclerotic parameters in HeFH compared to the HoFH patient group. Primary outcomes assessed relative to baseline values were as follows:

- Change in lipoprotein cholesterol (LDL-C),
- Change in total cholesterol,
- Change in proprotein convertase subtilisin/kexin type 9 (PCSK9) levels,
- Change in apolipoprotein-B (APO-B), and
- Change in non-high-density lipoprotein cholesterol (non-HDL-C).

The extracted data are available in Table 3.

**Table 3:** Data extracted from individual studies.

	Outcomes mean (SD or 95% CI)				
Trial name	Relative change in apolipoprotein-B from baseline (%)	Relative change in non-HDL from baseline (%)	Relative change in PCSK9 from baseline (%)	Relative change in total cholesterol from baseline (%)	Relative change in LDL-C from baseline (%)
ORION 9	−33.14 (−35.91 to −30.36)	−34.93 (−38.46 to −31.40)	−60.68 (−64.40 to −56.96)	−25.11(−27.83 to −22.39)	−41.15 (−44.52 to −37.77)
ORION 15	−46.81 (13.968)	−54.04 (14.230)	−75.97 (9.885)	−35.46 (11.941)	−56.3 (−61.1 to −51.4)
ORION 2	−25.0 (14.50)	−19.7 (13.93)	−59.0 (16.46)	−19.8 (13.68)	−20.96 (−49.97 to 8.05)
ORION 5	−5.8 (−15.9 to 4.3)	−6.2 (−17.1 to 4.7)	−53.1 (−70.2 to −35.9)	−4.9 (−14.3 to 4.5)	−7.2(−19.0 to 4.6)

### Data synthesis and statistical analysis

Data extraction was performed by two independent authors (S.S. and M.R.) using a standardized Excel spreadsheet. Disagreements were resolved following a discussion with the third author (C.R.W.). Outcomes for each trial included the patient population, degree of change in each outcome, year of publication, sample size, and first author. Data from four distinct clinical trials were analyzed. Only when at least two trials reported an outcome did we pool the meta-analysis data for that outcome. Statistical analysis was performed using OpenMeta Analyst Software. If data were reported as the mean (95% confidence interval), we converted them to the mean and standard deviation using the Cochrane formula to convert data to the mean and standard deviation^[6]^. The pooled mean changes were estimated for changes in PCSK9, apolipoproteins, non-HDL, LDL, and total cholesterol using the mean difference (MD) using the Mantel–Haenszel test, with a 95% confidence interval (CI) for continuous variables. These pooled mean changes were statistically compared between the HeFH and HoFH groups to identify any significant difference. Only variables that were reported in at least two studies were analyzed. To get the pooled estimates and 95% confidence interval, the random-effects model was employed. Heterogeneity among the studies was shown by the I^2 index. Mild heterogeneity was indicated by an I^2 value between 25% and 50%, moderate heterogeneity was indicated by a value between 50% and 75%, and severe heterogeneity was defined as a value larger than 75%. Potential publication bias was assessed using Begg’s correlation test. *P*-value < 0.05 indicated the primary outcomes to be significantly different between HeFH and HoFH groups.

### Risk of bias assessment

Two authors (M.J and R.K.) independently assessed the quality of all articles included in the review. The risk of bias was assessed with the “Revised Cochrane risk-of-bias tool for randomized trials” (RoB 2)^[7]^. We assessed for bias arising from the randomization process, bias due to deviations of intended interventions, bias due to missing outcome data, bias in outcome measurements, and bias in the selection of reported results. Individual domains of risk of bias and studies could be characterized as “unknown risk,” “low risk,” or “high risk.” There was no small study effect in trials except for ORION 2.

## Results

### Search strategy

**Figure 1.**
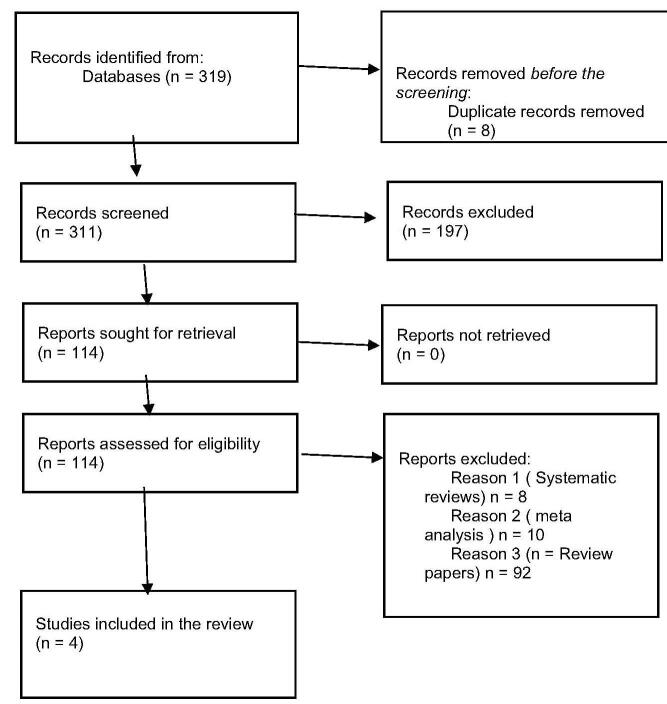
PRISMA flow diagram.

Three databases yielded a total of 319 articles, as can be seen in the PRISMA flow chart (Fig. 1). Eight of those items were disregarded due to duplication. An eligibility screening was conducted for the remaining 311 studies. After screening for titles and abstracts, 114 studies were found to be possibly eligible, and 114 studies with familial hypercholesterolemia either heterozygous or homozygous that met the criteria for full-text evaluation were chosen following a full-text assessment. Four clinical trials were included in this study after taking the inclusion criteria into account: ORION 2 (NCT02963311), ORION 5 (NCT03851705), ORION 9 (NCT03397121), and ORION 15 (NCT04666298). These studies can be traced by their clinical trial numbers.

**Figure 2.**
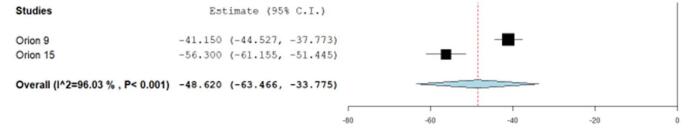
Percentage change in LDL-C from baseline in HeFH.

### Study characteristics

All the studies defined HeFH and HoFH as heritable conditions with patients having single and double-mutant alleles resulting in hypercholesterolemia, respectively. Each study described the subcutaneous injection of 300 mg of inclisiran and was of a clinical trial design. The study characteristic table provides more information on the type of familial hypercholesterolemia that was included, the number of cases in each study, and the reported outcomes (Table 1). The inclusion and exclusion criteria of each study are given in Supplemental Table S1 (http://links.lww.com/MS9/A718). Inclisiran sodium 300 g was given subcutaneously to enrolled patients in all the clinical trials, and values of atherosclerotic parameters were noted at the beginning of the trial and follow-up. Our studies provided the mean change (%) values at follow-up calculated relative to the baseline values. The study characteristics are available in Table 1.

**Table 1:** Characteristics of included studies.

Trial name	Study year	FH type	Population receiving drug *N*	Age [mean (SD)]	Number of males	Number of females	Investigated outcomes and variables
ORION 9	2019	Heterozygous	242	54.4 (12.48)	112	130	Change in apolipoprotien-B from baseline (%)
							Change in non-HDL from baseline (%)
							Change in PCSK9 from baseline (%)
							Change in total cholesterol from baseline (%)
							Absolute change in LDL from baseline
ORION 15	2022	Heterozygous	99	63.6 (10.46)	77	22	Change in apolipoprotien-B from baseline (%)
							Change in non-HDL from baseline (%)
							Change in PCSK9 from baseline (%)
							Change in total cholesterol from baseline (%)
							Absolute change in LDL from baseline
ORION 2	2018	Homozygous	4	37.0 (13.04)	2	2	Change in apolipoprotien-B from baseline (%)
							Change in non-HDL from baseline (%)
							Change in PCSK9 from baseline (%)
							Change in total cholesterol from baseline (%)
							Absolute change in LDL from baseline
ORION 5	2021	Homozygous	37	N/A	14	23	Change in apolipoprotien-B from baseline (%)
							Change in non-HDL from baseline (%)
							Change in PCSK9 from baseline (%)
							Change in total cholesterol from baseline (%)
							Absolute change in LDL from baseline

**Table 2:** Pooled proportions and confidence intervals.

Variable	Heterozygous				Homozygous				*P*- value
	Number of studies	Number of patients	Pooled proportion (95% CI) or mean (95% CI)	*I*^2^	Number of studies	Number of patients	Pooled proportion (95% CI) or mean (95% CI)	*I*^2^	
Relative change in LDL-C from baseline (%)	2	299	−48.62 (−63.5, −33.8)	96.03	2	41	−9.12 (−20.07, 1.77)	0	0.04
Relative change in total cholesterol from baseline (%)	2	299	−30.31 (−40.45, −20.16)	96.83	2	41	−11.50 (−26.01, 3.01)	69.25	0.03
Relative change in PCSK9 from baseline (%)	2	299	−68.41 (−83.39, −53.43)	98.04	2	41	−56.25 (−68.03, −44.46)	0	0.2
Relative change in apolipoprotein-B from baseline (%)	2	299	−39.97 (−53.37, −26.58)	97.85	2	41	−14.68 (−33.44, 4.09)	79.01	0.03
Relative change in non-HDL from baseline (%)	2	299	−44.51 (−63.24, −25.79)	98.54	2	41	−12.22 (−25.38, 0.93)	57.53	0.005

### Analysis of primary outcomes following drug administration

**Figure 3.**
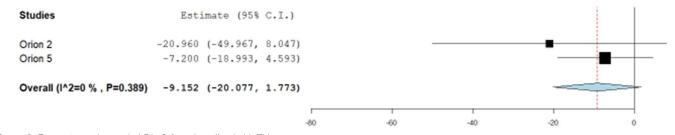
Percentage change in LDL-C from baseline in HoFH.

The mean change (%) in LDL-C relative to baseline in the heterozygous group was (heterozygous MD: –48.62, 95% CI: –63.46, –33.78) (Fig. 2) compared to the homozygous group (homozygous MD: –9.12, 95% CI: –20.07, 1.77) (Fig. 3).

**Figure 4.**
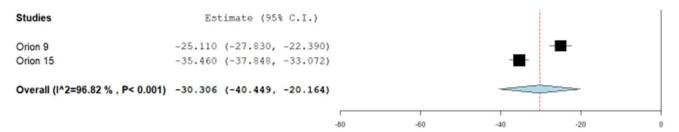
Percentage change in total cholesterol from baseline in HeFH.

**Figure 5.**
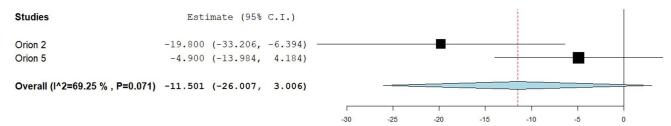
Plot showing change in total cholesterol from baseline in HoFH.

The mean change (%) in total cholesterol relative to baseline in the heterozygous group was (heterozygous MD: –30.31, 95% CI: –40.45, –20.16) (Fig. 4) as compared to the homozygous group (homozygous MD: –11.50, 95% CI: –26.01, 3.01) (Fig. 5).

**Figure 6.**
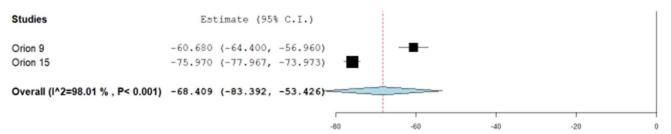
Percentage change in PCSK9 from baseline in HeFH.

**Figure 7.**
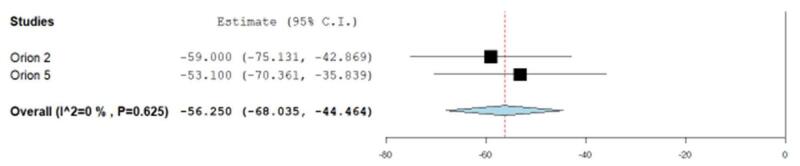
Plot showing change in PCSK9 from baseline in HoFH.

The mean change (%) in PCSK9 relative to baseline in the heterozygous group was (heterozygous MD: –68.41, 95% CI: –83.39, –53.43) (Fig. 6) as compared to the homozygous group (homozygous MD: –56.12 %, 95% CI: –67.66, –44.58) (Fig. 7).

**Figure 8.**
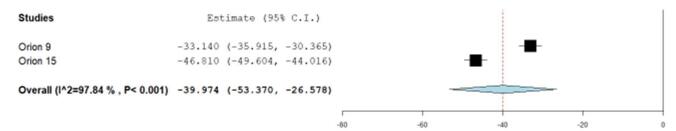
Percent change in apolipoprotein-B from baseline in HeFH.

**Figure 9.**
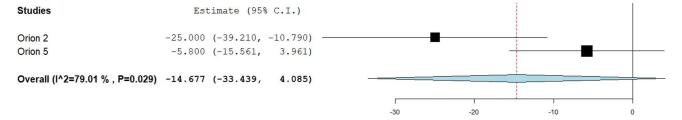
Plot showing change in apolipoprotein-B from baseline in HoFH.

The mean change (%) in apolipoprotein-B relative to baseline in the heterozygous group was (heterozygous MD: –39.97, 95% CI: –53.37, –26.58) (Fig. 8) as compared to the homozygous group (homozygous MD: –14.68, 95% CI: –33.44, 4.09) (Fig. 9).

**Figure 10.**
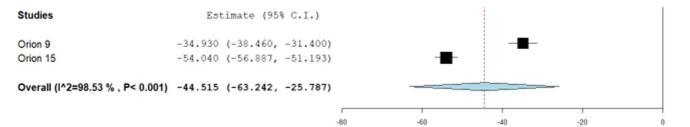
Percent change in non-HDL from baseline in HeFH.

**Figure 11.**
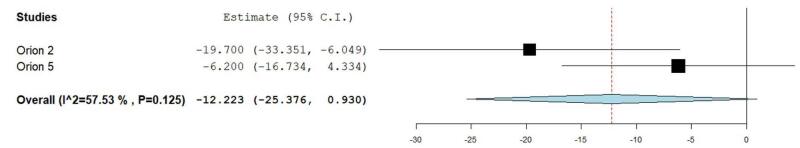
Plot showing change in non-HDL from baseline in HoFH.

The mean change (%) in the levels of non-HDL-C relative to baseline in the heterozygous group was (heterozygous MD: –44.51, 95% CI: –63.24, –25.79) (Fig. 10) as compared to the homozygous group (homozygous MD: –12.22, 95% CI: –25.38, 0.93) (Fig. 11).

**Figure 12.**
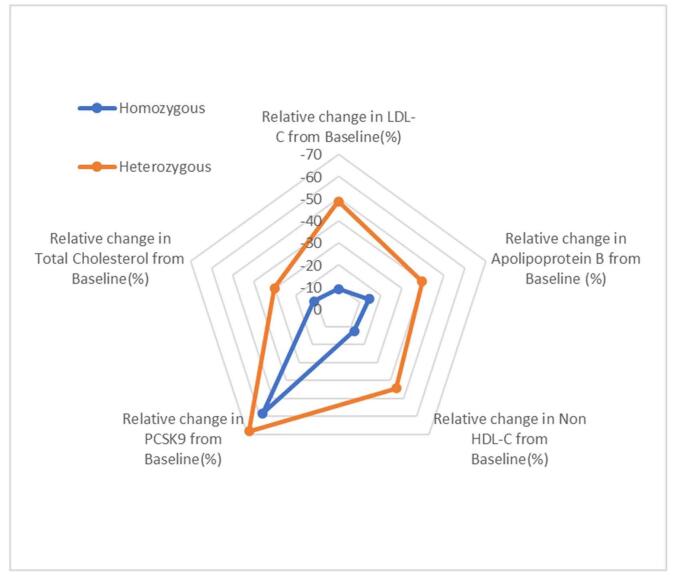
Radar plot showing pooled proportion of variables.

**Figure 13.**
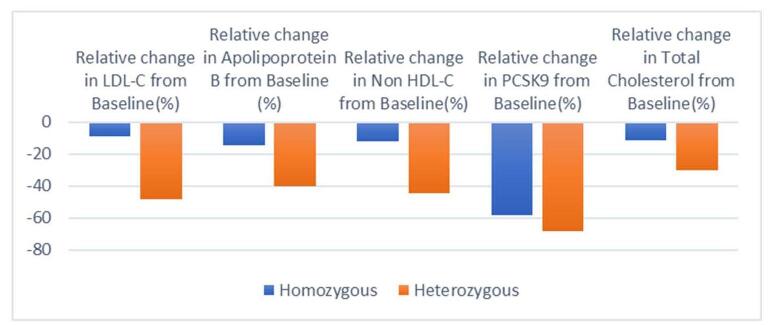
Bar charts show the results of the pooled proportion of variables.

The results of the pooled mean changes of variables are shown in Table 2. Figures 12 and 13 further illustrate the results.

## Discussion

Cardiovascular disease (CVD) is the leading cause of death in developed countries^[1]^. Atherosclerotic cardiovascular disease (ASCVD) is responsible for over 4 million deaths annually and accounts for up to 45% of all deaths^[3]^. Low-density lipoprotein cholesterol (LDL-C) is a significant risk factor for ASCVD in both primary and secondary prevention settings; thus, achieving and maintaining target LDL-C levels is crucial^[2]^. Given that a reduction of more than 50% in LDL-C is necessary to prevent ASCVD, lipid-lowering medications are the primary therapeutic approach for cardiovascular prevention^[3]^. FH is a prevalent genetic metabolic disorder associated with premature CVD^[3]^. Ezetimibe, due to its good tolerability, affordability, and oral administration, is recommended as a subsequent LDL-C-lowering therapy following statins. Despite this, many patients with HeFH continue to have LDL-C levels exceeding the recommended thresholds for primary and secondary prevention – 70 mg/dL and 100 mg/dL, respectively – indicating the need for additional therapy to further lower LDL-C as per current guidelines^[8]^. According to the 2018 American Heart Association (AHA)/American College of Cardiology (ACC)/Multi-Society guideline for managing blood cholesterol, a PCSK9 inhibitor is considered reasonable for patients with severe primary hypercholesterolemia who, despite statin and ezetimibe therapy, have LDL-C levels persistently above 100 mg/dL^[9]^. Additionally, PCSK9 inhibitors are recommended for patients with ASCVD undergoing secondary prevention when their LDL-C levels remain above 70 mg/dL despite receiving statin plus ezetimibe therapy^[9]^. Poor treatment adherence is a significant barrier to achieving LDL-C targets in patients at high risk of ASCVD. The asymptomatic nature of dyslipidemia often leads to decreased consistency in daily medication regimens, with many patients discontinuing their prescribed treatments within a year of initiation^[8]^.

Patients with HeFH have mutations in one allele of the LDL-C receptor, apolipoprotein-B (APO-B), or proprotein convertase subtilisin/kexin type 9 (PCSK9) genes^[8]^. In contrast, individuals with HoFH carry mutations in both alleles of these genes. HoFH is associated with a more severe clinical phenotype and poorer prognosis compared to HeFH due to the profound impairment in LDL-C receptor activity. Consequently, HoFH patients exhibit significantly higher LDL-C levels – often double those seen in HeFH patients – resulting in an increased risk of cardiovascular complications, sometimes occurring as early as the first decade of life^[5]^. Because most lipid-lowering therapies rely on residual LDL-C receptor activity, individuals with HoFH are typically resistant to statins and other standard lipid-lowering treatments. This resistance contributes to the worse clinical outcomes observed in HoFH compared to HeFH^[5]^.

Our research compares the changes in the levels of LDL-C, total cholesterol, PCSK9, non-HDL-C, and APO-B in patients having HoFH and HeFH, following the administration of 300 mg inclisiran sodium injections subcutaneously. This patient-level, pooled analysis from the ORION program demonstrates that inclisiran sodium administered subcutaneously in HoFH and HeFH populations has a different degree of effect on the atherosclerotic parameters in each population. In over 698 participants in the studies (ORION-9: 482, ORION-15: 156, ORION-2: 4, and ORION-5: 56), we found that the mean change in LDL-C (*P* < 0.05), total cholesterol (*P* < 0.05), non-HDL-C (*P* < 0.05), and in the levels of apolipoprotein (*P* < 0.05) was a significant different in HeFH relative to HoFH. However, we found an insignificant difference in the mean change in the levels of PCSK9 (*P* = 0.2). This is an important finding, as a primary target of inclisiran is the levels of PCSK9.

Our study clearly shows that the heterozygous population receives better reductions in LDL-C, total cholesterol, non-HDL-C, and apolipoprotein levels as compared to the homozygous population by the use of inclisiran sodium 300 mg. This is an important finding, as it attracts the curiosity of researchers to determine the factors behind the different responses of the drug in genotypically different populations. Clinically, physicians can determine heterozygous patients to be better suitable for this drug to get the best response.

The first method of inhibiting PCSK9 was to utilize monoclonal antibodies (mAbs), which specifically target PCSK9 receptors to stop them from attaching to LDL receptors^[10]^. Thus far, only two fully human monoclonal antibodies – evolocumab and alirocumab – have been authorized for use in clinical settings^[11]^. When administered subcutaneously either biweekly or monthly, evolocumab and alirocumab reduce LDL-C by 45 to 60%^[11]^. Even yet, it appears that the target demographic is comprised of patients who are at the highest and most extreme risk^[11]^. Sadly, due to a lack of head-to-head trials comparing inclisiran and anti-PCSK9 monoclonal antibodies, there are currently insufficient data to draw firm conclusions, which makes selecting the best course of treatment allocation difficult^[11]^.

On the other hand, the primary benefit of inclisiran compared to traditional pharmacotherapies and PCSK9 monoclonal antibodies lies in its less frequent dosing schedule – initially, three months, and then every 6 months. This schedule is expected to significantly enhance patient adherence, an improvement over daily oral medications, and more frequently administer monoclonal antibodies^[12]^. In clinical trials, inclisiran effectively lowered LDL-C levels by about 50% relative to the placebo. Although this reduction is somewhat less than that seen with monoclonal antibodies, the convenience of inclisiran’s dosing interval provides a notable advantage. Both therapy types offer substantial LDL-C reductions, yet questions about their long-term safety remain unanswered. The biannual administration of inclisiran could lead to better long-term compliance, a critical factor in managing cardiovascular risk. Nevertheless, the current lack of long-term cardiovascular outcome data is a significant gap; upcoming studies are essential to confirm whether the practical benefits of inclisiran translate into sustained safety and efficacy^[11]^

### Strengths and limitations

The thorough investigation and evaluation of the three sizable databases is this review’s strongest point. Compared to homozygous hypercholesterolemia, this is the first systematic review and meta-analysis comparing the effectiveness of inclisiran in patients with heterozygous for the condition. This research can contribute to further expansion in knowledge of the action of inclisiran at the genetic level.

Since we are aware of the limitations of our study, care should be taken when interpreting the findings. Every study that was included was conducted as a clinical trial. One of the main shortcomings of this systematic review is the heterogeneity in the follow-up periods. We were only able to incorporate some of the characteristics that we had planned to gather and stratify into the analysis because they had only been reported by a small number of researchers. Even though all of the included cases belonged to HeFH and HoFH, there was variation in their baseline lipid values, age, and ethnicity. The sample size of the ORION 2 trial was also small, leading to a small study effect in the pooled mean values.

Given that our data should be interpreted cautiously and that the cases are from tiny case reports and series from several centers, there was a significant amount of heterogeneity. Future multicenter studies must be carried out among centers with improved learning curves and that have homogenous patient selection, procedure timing, and technique protocols to get around some of the previously observed difficulties.

## Conclusion

This research highlights the lipid parameters that decrease significantly in heterozygotes as compared to homozygotes, with the administration of inclisiran. All the parameters considered in this research, LDL-C, total cholesterol, apolipoprotein-B, and non-HDL-C, decreased to a significantly greater degree in HeFH than HoFH, except for PCSK9. Further research in this aspect might reveal the causative factors behind this finding and widen the scope of our knowledge of the drug in this regard.

## Supplementary Material

## Data Availability

The data extracted from ORION trials and processed to produce the forest plots are publicly available on Clinicaltrials.gov and can be assessed to confirm the results of our research.

## Research registration unique identifying number (UIN)

The study is registered in Open Science Forum: https://doi.org/10.17605/OSF.IO/3Z8PV.
