# Supplementary material for: Efficacy and outcomes of inclisiran in the management of homozygous and heterozygous familial hypercholesterolemia: a systematic review and meta-analysis
Source: Ann Med Surg (Lond). 2025 Feb 6;87(3):1599–608. doi: 10.1097/MS9.0000000000002982 (PMC11981386; doi:10.1097/MS9.0000000000002982)
Supplement: Supplementary file 1 [file ms9-87-1599-s001.docx]

| Trial | Inclusion Criteria | Exclusion Criteria |
| --- | --- | --- |
| Orion 9 | Male or female participants ≥18 years of age.  History of HeFH with a diagnosis of HeFH by genetic testing; and/or a documented history of untreated LDL-C of >190 mg/dL, and a family history of familial hypercholesterolemia, elevated cholesterol or early heart disease that may indicate familial hypercholesterolemia.  Serum LDL-C ≥2.6 millimoles (mmol)/liter (L) (≥100 mg/dL) at screening.  Fasting triglyceride <4.52 mmol/L (<400 mg/dL) at screening.  Participants on statins should be receiving a maximally tolerated dose.  Participants not receiving statins must have documented evidence of intolerance to all doses of at least 2 different statins.  Participants on lipid-lowering therapies (such as a statin and/or ezetimibe) should be on a stable dose for ≥30 days before screening with no planned medication or dose change during study participation. | New York Heart Association (NYHA) class IV heart failure.  Uncontrolled cardiac arrhythmia  Uncontrolled severe hypertension  Active liver disease  Females who are pregnant or nursing, or who are of childbearing potential and unwilling to use at least 2 methods of highly effective contraception (failure rate less than 1% per year) (combined oral contraceptives, barrier methods, approved contraceptive implant, long-term injectable contraception, or intrauterine device) for the entire duration of the study. Exemptions from this criterion:  Women >2 years postmenopausal (defined as 1 year or longer since last menstrual period) AND more than 55 years of age.  Postmenopausal women (as defined above) and less than 55 years of age with a negative pregnancy test within 24 hours of randomization.  Women who are surgically sterilized at least 3 months prior to enrollment.  Males who are unwilling to use an acceptable method of birth control during the entire study period (condom with spermicide).  Treatment with other investigational products or devices within 30 days or 5 half-lives of the screening visit, whichever is longer.  Treatment (within 90 days of screening) with monoclonal antibodies directed towards PCSK9. |
| Orion 15 | Participants with history of CAD or participants categorized in 'high risk' by Japan Atherosclerosis Society (JAS) 2017 guidelines or participants with heterozygous familial hypercholesterolemia (HeFH)  As per the JAS 2017 guideline, participants not meeting the LDL-C management targets.  Participants on statins should be receiving a maximally tolerated dose.  Participants not receiving statins must have documented evidence of intolerance to at least one statin.  The lipid-lowering therapy should have remained stable for ≥ 30 days before screening with no planned medication/ dose change until Day 180 | Participants diagnosed with homozygous familial hypercholesterolemia (HoFH).  Treatment (within 90 days of screening) with monoclonal antibodies directed towards PCSK9.  New York Heart Association (NYHA) class IV heart failure or last known left ventricular ejection fraction <25%.  Cardiac arrhythmia within 3 months prior to randomization that is not controlled by medication or via ablation.  Uncontrolled hypertension: systolic blood pressure >160 mmHg or diastolic blood pressure >100 mmHg prior to randomization despite antihypertensive therapy.  Active liver disease defined as any known current infectious, neoplastic, or metabolic pathology of the liver or unexplained elevations in alanine aminotransferase (ALT), aspartate aminotransferase (AST), >3x the upper limit of normal (ULN), or total bilirubin >2x ULN at screening.  Severe concomitant non-cardiovascular disease that carries the risk of reducing life expectancy to less than 2 years. |
| Orion 2 | Males and females, ≥12 years of age with a diagnosis of homozygous familial hypercholesterolemia by genetic confirmation or a clinical diagnosis based on a history of an untreated low-density lipoprotein cholesterol (LDL-C) concentration >500 mg/deciliter (dL) [13 millimoles/liter (mmol/L)] together with either xanthoma before 10 years of age or evidence of heterozygous familial hypercholesterolemia in both parents.  Stable on a low-fat diet.  Stable on pre-existing, lipid-lowering therapies (such as statins, cholesterolabsorption inhibitors, bile-acid sequestrants, or combinations thereof) for at least 4 weeks with no planned medication or dose change for the duration of study participation.  Fasting central lab LDL-C concentration >130 mg/dL (3.4 mmol/L) and triglyceride concentration <400 mg/dL (4.5 mmol/L).  Body weight of 40 kilograms (kg) or greater at screening. | LDL or plasma apheresis within 8 weeks prior to the screening visit, and no plan to receive it during the study because of the attendant difficulty in maintaining stable concentrations of LDL-C while receiving apheresis.  Use of mipomersen or lomitapide therapy within 5 months of screening.  Previous treatment with monoclonal antibodies directed towards PCSK9 within 8 weeks of screening. |
| Orion 5 | Diagnosis of HoFH by genetic confirmation or a clinical diagnosis based on a history of an untreated LDL-C concentration >500 mg/dL (13 mmol/L) together with either xanthoma before 10 years of age or evidence of heterozygous familial hypercholesterolemia in both parents  Stable on a low-fat diet.  Subjects on statins should be receiving a maximally tolerated dose. Maximum tolerated dose is defined as the maximum dose of statin that can be taken on a regular basis without intolerable adverse events.  Subjects not receiving statins must have documented evidence of intolerance to at least two different statins.  Subjects on lipid-lower therapies (such as statin and/or ezetimibe) should be on a stable dose for ≥30 days before screening with no planned medication or dose change during study participation.  Fasting central laboratory LDL-C concentration ≥130 mg/dL (3.4 mmol/L).  Triglyceride concentration <400 mg/dL (4.5 mmol/L)  No current or planned renal dialysis or renal transplantation  Subjects on a documented regimen of LDL or plasma apheresis will be allowed to continue the apheresis during the study, if needed.  Subjects must be willing and able to give written informed consent before initiation of any study-related procedures. The subject should be willing to comply with all required study procedures.  Willing to follow all study procedures including adherence to dietary guidelines, study visits, fasting blood draws, and compliance with study treatment regimens. | Use of Mipomersen or Lomitapide therapy within 5 months of screening  Treatment (within 90 days of screening) with monoclonal antibodies directed towards PCSK9  New York Heart Association (NYHA) class IV heart failure or last known left ventricular ejection fraction <25%  Major adverse cardiovascular event within 3 months prior to randomization  Planned cardiac surgery or revascularization  Uncontrolled severe hypertension: systolic blood pressure >180 mmHg or diastolic blood pressure >110 mmHg prior to randomization despite anti-hypertensive therapy  Active liver disease defined as any known current infectious, neoplastic, or metabolic pathology of the liver or unexplained alanine aminotransferase (ALT), aspartate aminotransferase (AST), elevation >3x ULN, or total bilirubin >2x upper limit of normal (ULN) at screening confirmed by a repeat measurement at least 1 week apart  Severe concomitant noncardiovascular disease that carries the risk of reducing life expectancy to less than the duration of the trial  History of malignancy that required surgery (excluding local and wide-local excision), radiation therapy and/or commencement of systemic therapy as treatment during the 3 years prior to randomization  Females who are pregnant or nursing, or who are of childbearing potential and unwilling to use at least one acceptable effective method of contraception (eg, oral contraceptives, barrier methods, approved contraceptive implant, long- term injectable contraception, intrauterine device) for the entire duration of the study. Exemptions from this criterion:  Women >2 years postmenopausal (defined as 1 year or longer since their last menstrual period) AND more than 55 years of age  Postmenopausal women (as defined above) and less than 55 years of age with a negative pregnancy test within 24 hours of enrolment  Women who are surgically sterilized at least 3 months prior to enrolment  Known history of alcohol and/or drug abuse within 5 years  Any condition that according to the investigator could interfere with the conduct of the study, such as but not limited to:  Subjects who are unable to communicate or to cooperate with the investigator.  Unable to understand the protocol requirements, instructions and study-related restrictions, the nature, scope, and possible consequences of the study (including subjects whose cooperation is doubtful due to drug abuse or alcohol dependency)  Unlikely to comply with the protocol requirements, instructions, and study-related restrictions (eg, uncooperative attitude, inability to return for follow-up visits, and improbability of completing the study)  Have any medical or surgical condition, which in the opinion of the investigator would put the subject at increased risk from participating in the study  Persons directly involved in the conduct of the study  Any uncontrolled or serious disease, or any medical or surgical condition, that may either interfere with participation in the clinical study, and/or put the subject at significant risk (according to investigator's [or delegate] judgment) if he/she participates in the clinical study  Any underlying known disease, or surgical, physical, or medical condition that, in the opinion of the Investigator, might interfere with the interpretation of clinical study results  Treatment with other investigational medicinal products or devices within 30 days or 5 half-lives of the screening visit, whichever is longer  Previous participation in the study  Hypersensitivity to any of the ingredients of Inclisiran |

S1. Inclusion and exclusion criteria
